# Pigeon fanciers lung: a case report

**DOI:** 10.1186/1757-1626-1-37

**Published:** 2008-07-15

**Authors:** PA Mehta, P Wills, SK Kohli, SW Dubrey

**Affiliations:** 1 Clinical Cardiology, National Heart & Lung Institute, Imperial College, Dovehouse Street, London, SW3 6LY, UK; 2 The Hillingdon Hospital, Pield Heath Road, Uxbridge, UB8 3NN, UK

## Abstract

**Introduction:**

Pigeon fanciers lung, a form of hypersensitivity pneumonitis, is an unusual but important occupational and recreational cause of severe and debilitating breathlessness.

**Case presentation:**

We report the case of a 61-year-old Caucasian man suffering with severe breathlessness due to pigeon fanciers lung. He had previously raced and bred pigeons for 25 years but had discontinued the pursuit a decade ago.

**Conclusion:**

Pigeon fanciers lung can be associated with severe debilitating dyspnoea and patients may present many years after exposure to avian antigens. Physicians should be encouraged to take a detailed occupational and recreational history in any patient presenting with unexplained breathlessness.

## Case report

In the United Kingdom, it is estimated that as many as 100,000 people breed and race pigeons. We present the case of a patient with pigeon fanciers lung, resulting in severely debilitating chronic breathlessness.

A 61-year-old man was admitted with a 15-year history of progressively worsening dyspnoea, dry cough and fatigue. The patient had been dyspnoeic at rest for the past six months. He denied any history of chest pain or peripheral oedema. The patient was known to be hypertensive treated with Amlodipine. He was a life-long non-smoker and denied any exposure to asbestos or significant family history. He had previously bred and raced pigeons for 25 years but had abandoned this pursuit 10 years previously. Five years later he had noticed the start of his breathlessness.

On clinical examination he was apyrexial, pulse 110 beats per minute, blood pressure 136/72 mmHg and tachypnoeic at 25 breaths per minute. Oxygen saturations were 85% on 60% oxygen. Chest auscultation revealed bilateral basal and mid zone inspiratory crackles with no wheeze.

Full blood count, serum electrolytes, renal, hepatic and thyroid function were normal. C-reactive protein was 9 mg/L. An electrocardiogram was unremarkable. Arterial blood gases on 60% oxygen showed type I respiratory failure: pH of 7.45 (7.35–7.45), pCO2 4.98 kPa (4.67–6.4), pO2 6.11 kPa, bicarbonate 25.8 mmol/L and base excess 1.7.

**Figure 1:**
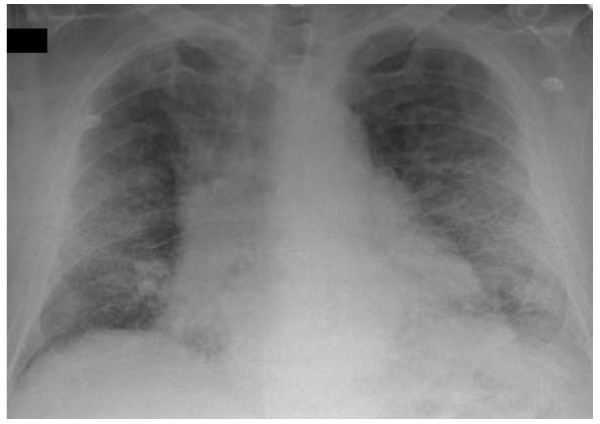
Chest radiograph showing bilateral interstitial reticular shadowing with loss of lung volume.

The chest radiograph (Figure 1) demonstrated bilateral interstitial reticular shadowing with loss of lung volume.

**Figure 2:**
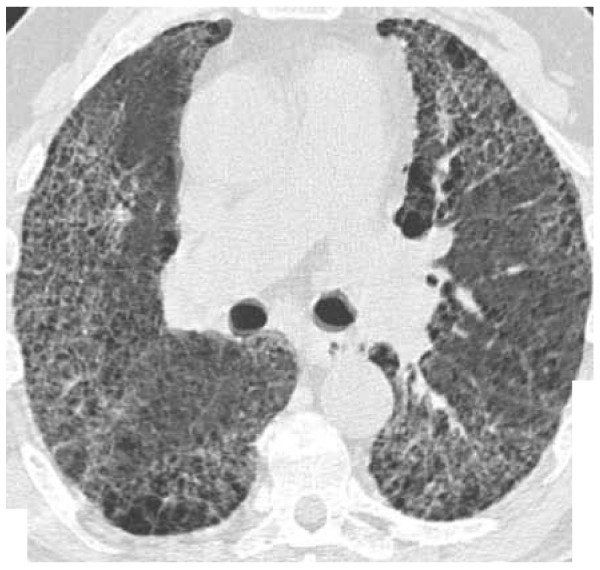
Computed tomography scan of the thorax showing extensive honeycombing in both lungs with widespread ground glass opacification and patchy asymmetrical areas of normal lung tissue.

A high-resolution computed tomography scan (Figure 2) of the thorax showed extensive honeycombing in both lungs with widespread ground glass opacification and patchy asymmetrical areas of normal lung tissue. The appearances are consistent with a longstanding allergic alveolitis.

The working diagnosis was hypersensitivity pneumonitis (extrinsic allergic alveolitis). On further assessment he was found to be positive for anti-avian antibodies confirming a diagnosis of pigeon fanciers lung. Treatment included oxygen and oral corticosteroids although there was no clinical response to the steroids, which were therefore discontinued.

## Discussion

Hypersensitivity pneumonitis (HP) is a collection of allergic lung diseases caused by the inhalation of antigenic organic particles or fumes [1,2]. HP is histologically characterised by the triad of non-necrotising granulomas, chronic inflammatory change in small airways and diffuse interstitial infiltrates of chronic inflammatory cells [3,4].

Pigeon fanciers lung is a type of HP caused by airborne exposure to avian antigens [1,2,5]. The disease may present acutely or sub-acutely and such episodes usually resolve with cessation of the antigen exposure. Chronic disease may progress to irreversible disease [5].

Lifestyle questions are integral to a case of this type and must include an assessment of potential hazards from occupational and animal exposure. Prevention and early diagnosis of those at risk of developing chronic lung disease requires adequate knowledge, awareness and understanding of the disease in the enthusiast and by the medical professional.

Corticosteroids are indicated for the treatment of severe acute and sub-acute HP and for chronic HP that is severe or progressive. Long-term corticosteroid therapy for the treatment of chronic HP should be considered only if objective improvements in clinical signs, pulmonary function, or radiographic abnormalities are documented [5].

## Acknowledgements

Written informed consent was obtained from the patient for publication of this case report and accompanying images. A copy of the written consent is available for review by the Editor-in-Chief of this journal.
